# Proceedings of the Conference “CMV Vaccine Development—How Close Are We?” (27–28 September 2023)

**DOI:** 10.3390/vaccines12111231

**Published:** 2024-10-29

**Authors:** Mark R. Schleiss, Chelsea M. Crooks, Krithika P. Karthigeyan, Rebecca M. Kruc, Claire E. Otero, Hsuan-Yuan (Sherry) Wang, Sallie R. Permar, Stanley A. Plotkin, Rajeev Gautam

**Affiliations:** 1 Division of Infectious Diseases, Department of Pediatrics, University of Minnesota Medical School, 2001 6th Street SE, Minneapolis, MN 55455, USA; 2 BB-869-H, Belfer Research Building, Weill Cornell Medicine, 413 East 69th Street, New York, NY 10021, USA; cmc4008@med.cornell.edu (C.M.C.); kpk4001@med.cornell.edu (K.P.K.); clo4001@med.cornell.edu (C.E.O.);; 3 Department of Pediatrics, Mayo Clinic, 200 First Street SW, Rochester, MN 55905, USA; kruc.rebecca@mayo.edu; 4 Department of Pediatrics, Weill Cornell Medicine, 1300 York Ave Box 65, New York, NY 10065, USA; sap4017@med.cornell.edu; 5 Perelman School of Medicine, University of Pennsylvania, 3400 Civic Center Boulevard, Building 421, Philadelphia, PA 19104, USA; 6 Program Officer at Virology Branch, Division of Microbiology and Infectious Diseases, NIAID, NIH, 5601 Fisher’s Lane, Rockville, MD 20892, USA; rajeev.gautam@nih.gov

**Keywords:** congenital cytomegalovirus (CMV), CMV vaccine, CMV advocacy, CMV immunity

## Abstract

Congenital cytomegalovirus (cCMV) is the most common infectious cause of disability in children, including sensorineural hearing loss. There is interest in developing a pre-conception vaccine that could confer protective immunity on a woman of child-bearing age, hence resulting in a reduced cCMV disease burden. Other populations, including solid organ transplant (SOT) and hematopoietic stem cell transplant (HSCT) patients, could also benefit from CMV vaccination. To review and discuss vaccines that are in clinical development, a workshop, sponsored by the National Institutes of Health (NIH) and the National Institute of Allergy and Infectious Diseases (NIAID), was empaneled. At this workshop, correlates of protective immunity against CMV, epidemiologic features of CMV transmission, and vaccine platforms in development were reviewed. Representatives from academia, pharma, and the NIH engaged in discussion on the current state-of-the-art in CMV vaccinology. A summary of the presentations from this is provided in this report.

## 1. Introduction

A vaccine against human cytomegalovirus (CMV) infection is a major public health priority. Such a vaccine could be employed not only to help prevent or ameliorate CMV disease in the setting of SOT or HSCT, but also to address the more compelling need to lessen the severity of pediatric disabilities caused by cCMV infection. Since cCMV is the most common infectious cause of disability, including sensorineural hearing loss (SNHL), in children, the need for the development of a pre-conception vaccine is compelling.

Toward the goal of providing a state-of-the-art update on CMV vaccines, an NIAID Workshop, “CMV Vaccine Development—How Close Are We?”, was held from Wednesday, 27 September 2023 to Thursday, 28 September 2023, at the Grand Hall (NIAID Conference Center), 5601 Fishers Lane, Rockville, MD 20852, USA. Numerous candidate vaccines that are in various stages of clinical development were discussed. The purpose of the meeting was to exchange information among expert representatives from academia, industry, and federal agencies on the current status of, and the challenges to, development and licensure of a CMV vaccine.

This conference report represents the work of several “rapporteurs”—trainees from the University of Minnesota and Weill-Cornell Medical Schools who transcribed the presentations and prepared the written summaries. There were four separate sessions at the workshop: (1) cCMV epidemiology; (2) CMV virology and vaccine targets; (3) immune correlates of protection; and (4) current vaccines in clinical trials/clinical development. A synopsis of each session is provided in this manuscript, and the primary correspondent for each session summary is indicated.

## 2. Session 1: Epidemiology of Congenital CMV and Target Populations for Vaccination (R. Kruc)

This session provided an overview of CMV epidemiology, disease course, and the impact of infection on immunity, pathogenesis, and populations affected. The unique epidemiology of cCMV in different populations was reviewed. The pathogenesis of transmission from mother to fetus was discussed, including challenges that exist with respect to testing during pregnancy.

A key point emphasized in the session was that maternal CMV immune status has an effect on CMV transmission and sequelae in the fetus. Although vaccination programs have emphasized pre-conception immunization of adolescents and young women, the mathematical modeling of the potential benefit of CMV vaccination in young children was presented. Parallels between cCMV and CMV in SOT recipients were discussed, and vaccine trials in SOT patients were reviewed. Data regarding the role of CMV as an immunomodulator in HIV-positive patients with tuberculosis in Uganda were also presented, toward the goal of developing vaccines for these individuals.

**Speaker 1: Dr. Stanley Plotkin**

Dr. Plotkin introduced the biology and reviewed the historical context of cCMV infections, emphasizing the importance of developing a CMV vaccine. He discussed that CMV is a concern for both seronegative and seropositive women during pregnancy, and that infection and sequelae risk vary throughout the course of a pregnancy [1,2]. Dr. Plotkin pointed out other reasons why a CMV vaccine is important, such as the fact that CMV stands out as the most important infectious complication of SOT/HSCT, and there is some evidence that CMV has oncogenic properties, including tumors such as glioblastoma multiforme and childhood acute lymphocytic leukemia. He introduced possible target populations for a CMV vaccine, including infants, adolescents, women, and transplant recipients. Dr. Plotkin noted that there are many important considerations in thinking about CMV vaccines: age of administration, breadth across strains, consideration of the correlates of protection, demonstration of the duration of protection after vaccination, and protection of both seropositive and seronegative women against infection during pregnancy.

Key Points:

- CMV is an infection that has significant morbidity during pregnancy and in SOT recipients.
- It is important from a public health perspective to pursue a CMV vaccine; there are many considerations in how to make a CMV vaccine the most successful, including vaccine design, targets of the immune response, and implementation into clinical practice, and these strategies may differ depending on the target population (transplant patients, or women of child-bearing age).

**Speaker 2: Dr. Sallie Permar**

Dr. Permar provided an overview of the remaining gaps in knowledge that need to be addressed for the development of a CMV vaccine. There have been over 50 years of work on CMV vaccine development, yet we are still awaiting an approved vaccine. She emphasized that there are eight CMV vaccine platforms in phase 1 and 2 trials. Remaining knowledge gaps that were discussed included: (1) an incomplete definition of the immunologic correlates of protection; (2) the need to understand the prevention of mucosal acquisition of virus; (3) the mechanisms of congenital transmission; and (4) the host and viral factors that attenuate viremia and CMV end-organ disease in the transplant setting. Pre-existing immunity during pregnancy is at least partially protective against vertical transmission, and passive CMV-specific antibody (CMV-Ig) is protective against CMV disease in SOT patients. Considerations also include the fact that neutralizing antibodies versus other vaccine responses (such as T cell responses) represent different mechanisms of protection, and need to be studied separately. Dr. Permar commented on successes observed in vaccine efficacy trials performed to date. For example, two recombinant glycoprotein B [gB] vaccine trials with adjuvant MF59 (gB/MF59) achieved ~50% efficacy rates against the acquisition of primary infection in seronegative recipients [3,4]. Antigen choice is another consideration, and potential candidates include entry and non-entry glycoproteins, T cell targets, and virally encoded immune evasion molecules. The CMV pentameric complex (PC), gB, and the gH/gL/gO trimer are entry glycoprotein candidates for CMV vaccines, and gB and PC are in clinical trials. Non-entry glycoproteins are targets of antibody-dependent cellular cytotoxicity (ADCC) and include CMV gene products UL141 and UL16. Both CD8+ and CD4+ T cell responses contain CMV viral replication; viral evasion of CD8+T cells may potentiate replication. The impact of CMV-encoded immune evasion molecules was considered, including virally encoded chemokines and G-protein coupled receptors [5], which may be important considerations in vaccine design. Antigen design considerations include the targeting of specific virally encoded epitopes, induction of both neutralizing and non-neutralizing antibodies, the importance of conservation of target CMV proteins across strains, the importance of recapitulating the “natural” conformation of proteins (e.g., the prefusion conformation of gB), and consideration of the B cell lineage of host responses [6,7,8,9].

Key Points:

- Next-generation CMV vaccine design achieving >50% efficacy is a goal that might be achievable using current vaccines that elicit anti-gB antibody response.
- Use of conformation-appropriate glycoproteins may be key to protection.
- This antibody response may not primarily work through the induction of viral-neutralizing antibodies.

**Speaker 3: Dr. Tatiana Lanzieri**

Dr. Lanzieri of the Centers for Disease Control and Prevention gave an overview of CMV epidemiology. Dr. Lanzieri noted that cCMV occurs in 4.5 per 1000 live births in the US, which equates to 16,000 infected newborns in 2020. It is estimated that 0.5% of cases result in a fatal outcome, but most cases have no long-term health effects in the congenitally infected newborn [10]. Dr. Lanzieri noted that 5–10% of cases of prelingual SNHL are caused by cCMV, and up to half of such cases of pediatric SNHL that occur in early childhood are delayed in onset, and hence not identified by newborn hearing screen. Minnesota has cCMV universal newborn screening, and New York state has commenced an NIH-funded universal cCMV screening program. Connecticut has also passed a legislative mandate to begin cCMV universal screening by 2025.

CMV seroprevalence in the general population is variable. Globally, seroprevalence ranges from 25 to 95% depending on geographic location and other factors, including race, ethnicity, and socio-economic status [11]. It was emphasized that, for much of the world, we do not know the seroprevalence of CMV. Factors associated with differences in cCMV prevalence included race/ethnicity, maternal age, socioeconomic status, birth order, interpregnancy interval, amount of contact with children, and maternal seroprevalence. Dr. Lanzieri commented on the factors impacting cCMV prevalence. It was noted that the CMV and Hearing Multicenter Screening Study (CHIMES) study found the highest prevalence by race/ethnicity among Black people, and a lower prevalence in Hispanic white, non-Hispanic white, and Asian people [12]. Other studies have noted that CMV seroprevalence differences by race as well, although this varies across states. The average age of mothers at first birth is widely different depending on the state, size of city, and rural/urban divide. Young maternal age is a risk factor for delivering a baby with cCMV [13]. CMV primary infection prevalence among adults includes a 2.3% prevalence among pregnant people, a 9% prevalence among daycare providers, a 24% prevalence among parents of children shedding CMV [14], and a 0.35% prevalence among pregnant people screened by 23 weeks gestational age in the CMV-Ig clinical trial [15]. Overall CMV seroprevalence among women aged 20–49 years in the USA between 1999 and 2004 was >60% but higher with age and number of live births, lower in non-Hispanic whites (50%) than in other groups (85%), and higher with family income below the poverty threshold, or with more crowded housing and no insurance [16].

Next, Dr. Lanzieri noted that the risk of delivering a newborn with cCMV is four-fold higher for seronegative versus seropositive women if there is an exposure to CMV during pregnancy. It was also emphasized that most babies with cCMV are, in fact, born to women with pre-conception immunity (seropositives). cCMV prevalence is nearly two-fold higher among second-born children compared to first-born, and there is a higher risk of cCMV infection associated with a shorter interpregnancy interval. Young children with CMV infection rarely have symptoms, but do have high amounts of viral shedding in saliva and urine; young children also become infected through breastfeeding, and via child-to-child and child-to-adult transmission (particularly in the context of young children attending group day-care).

Dr. Lanzieri highlighted strategies to reduce the burden of cCMV. These include preventing infection during pregnancy by education to reduce CMV exposures among pregnant persons, and potentially (in the future) by vaccination; by preventing transmission of infection to the fetus through testing for primary maternal CMV infection during pregnancy (and in some cases by offering therapy with high-dose valacyclovir); and by enhanced detection of cCMV infection through newborn screening. Universal cCMV screening offers promise of early detection of, and intervention for, SNHL. Interventions could include antiviral treatment for symptomatic infants (Table 1). CDC activities include measuring CMV seroprevalence among children 1–5 years of age as part of the 2022–2023 National Health and Nutrition Examination Survey (NHANES) dataset, a cCMV surveillance pilot testing project through SET-NET with eight jurisdictions in order to monitor trends and identify groups at higher risk of cCMV and inform vaccination and newborn screening policy, and continued development and evaluation of dried blood spot PCR for newborn cCMV screening.

Key Points:

- The epidemiology of cCMV varies across the United States and the world, and is influenced by a variety of maternal, racial, and socioeconomic characteristics, as well as environmental factors.
- There are multiple epidemiologic factors during pregnancy that influence the likelihood of an infant being affected by cCMV.
- There are multiple potential avenues to prevent the transmission of CMV to the fetus, and one important future consideration is the development of an effective vaccine.

**Speaker 4: Dr. Karen Fowler**

Dr. Fowler spoke on cCMV transmission in seronegative and seropositive women. CMV transmission to the fetus is not straightforward. Dr. Fowler emphasized that maternal CMV infections before and during pregnancy contribute to fetal infections, but most women and their healthcare providers have little CMV awareness, and likely do not know their CMV status before or during pregnancy. National OB/GYN organizations, such as the American College of Obstetrics and Gynecology (ACOG) do not recommend routine maternal CMV screening, the rationale being that knowledge of maternal CMV serostatus does not predict risk nor inform management [17]. Primary CMV maternal infection during pregnancy necessitates serologic testing before 14 weeks to identify a primary infection occurring before 3 months gestational age. Another strategy is the detection of DNAemia; the presence of CMV DNA in maternal bodily fluids can supplement information from serologic diagnosis, although it does not prove fetal infection. Confirmation of cCMV infection as a prenatal diagnosis requires detection of CMV PCR in amniotic fluid (amniocentesis > 21 weeks’ gestation and > 6 weeks after estimated maternal seroconversion), and in the newborn by a positive CMV PCR in urine (alternatively, in blood) in the first 21 days of life. Identification of CMV DNA in the newborn dried blood spot (DBS) is gaining traction as an acceptable diagnostic test. Saliva may also be used, although care should be taken to consider that breast-feeding can lead to false positive tests due to the presence of CMV DNA in colostrum and/or breast milk.

Next, Dr. Fowler spoke about challenges with diagnosing CMV infection during pregnancy [18]. The CMV-Ig trial found that amniocentesis accurately predicted cCMV-positive status, but was not predictive of the severity of infection and symptom status [19]. Amniotic fluid viral load is an independent predictor of the severity of anomalies and is correlated with fetal MRI results. The timing of cCMV infection during pregnancy affects fetal transmission rate, fetal insults, and the likelihood of symptoms at birth. Dr. Fowler also noted that transmission rates increase across the trimesters of pregnancy [20], but the magnitude of the fetal insult and the probability of symptomatic disease at birth decreases if infection occurs later in gestation.

Non-primary CMV infection (reactivation or secondary infection) is difficult to measure, but more recent studies have demonstrated more than one viral strain can be present in both the maternal and fetal/newborn compartments. Seropositive women are frequently reinfected with multiple strains. Diagnosing non-primary CMV infection is therefore quite challenging, since sero-status does not define the presence or absence of reinfection, at least not with readily available commercial assays. Among women seropositive before pregnancy, the risk of CMV vertical transmission is variably reported to be in the 1–3% range [21]. The reported proportion of symptomatic cCMV in neonates born in highly seropositive populations is highly variable, but may be as high as 23.9% [22]. The key point is that non-primary infections account for a large number of cCMV infections. Dr. Fowler noted that we have information on transmission rates in the setting of primary maternal CMV infections in pregnancy, but we do not know the prevalence of maternal nonprimary infections [23], largely because such infections are so difficult to document.

Key Points:

- Screening mothers for CMV during pregnancy is not routinely conducted in the US and there are no consensus recommendations from ACOG supporting this.
- It remains uncertain whether cCMV transmission in CMV seropositive women is associated with CMV reactivation or reinfection with a new strain, but either mechanism is plausible, and vertical transmission probably occurs by both mechanisms.

**Speaker 5: Dr. Soren Gantt**

Dr. Gantt discussed CMV transmission among children and the possibility of targeting toddlers as a vaccination population. CMV is transmitted through breast milk, saliva, urine, sex, blood, and transplacental transmission. Young CMV-infected children are super-spreaders; they are highly infectious and shed high viral loads in urine and saliva for prolonged periods. Reinfection of women by infants with high viral load shedding (from a study in Uganda) demonstrated that 88% later had evidence of reinfection, and the risk of maternal reinfection was directly proportional to infant saliva viral load [24]. This was not seen with older siblings in the same household, presumably because they were shedding reduced amounts of virus. Dr. Gantt noted that this observation potentially drives optimism for a vaccine to prevent cCMV via the prevention of household spread through mucosal acquisition. Unknown questions still include what vaccine efficacy level against primary infection is required to prevent cCMV in the population, and who should be vaccinated.

Dr. Gantt noted that most models have indicated the greatest potential benefit of a CMV vaccine may be realized by vaccinating young children. His team used mathematical modeling of CMV transmission in an agent-based stochastic model which tracked the dynamics of each individual within a population [25]. This model was found to fit the existing epidemiologic data well. The model accurately predicted primary rates of CMV infections during pregnancy and the predictions fit existing NHANES data. Estimates of natural immune protection from the model include the observation that antiviral immunity against a new infection wanes over time with a half-life of ~9 months; there is a 36% lower risk of reinfection than primary infection given the same level of exposure; and viral shedding following reinfection was 66% lower than that observed in primary infection. Models comparing a “sterilizing immunity vaccine” and a “natural immunity vaccine” showed that the greatest reduction in cCMV could be conferred by vaccinating infants (by preventing transmission to women); indeed, there are multiple models that support the idea that vaccinating infants with a sterilizing vaccine prevents more CMV infections overall when compared to vaccinating individuals in other age groups. Dr. Gantt’s conclusions include the following: (1) infant vaccination appears to have the greatest degree of impact on cCMV, by reducing transmission to pregnant women; (2) future CMV vaccine trials should include young children and should evaluate transmission as well as acquisition; and (3) even modestly protective vaccines (e.g., gB/MF59) [3,4] and the replication-disabled CMV vaccine (V160) [26] might be effective at the population level if given to infants. Dr. Gantt also noted that infant CMV vaccination posed challenges, including acceptability concerns about the lack of direct benefits for the child, determination of the most appropriate clinical trial endpoints, and gauging the route to regulatory approval.

Key Points:

- Dr. Gantt’s team’s research used mathematical modeling of CMV transmission to demonstrate the benefits of vaccinating young children.
- Most models show that a sterilizing vaccine given to young children prevents the most CMV infections compared to vaccinating other ages of people.

**Speaker 6: Dr. Marianne Leruez-Ville**

Dr. Leruez-Ville spoke on CMV infections in populations of seronegative and seropositive French women, from the perspective of a European researcher (Université Paris Cité). There is a pooled prevalence of cCMV of 0.45% across Europe. Primary and non-primary maternal infections each account for about half of infections in their studies. Seronegative women had a 3–4 times higher risk for cCMV infection in the infant than seropositive women. The seroconversion rate in pregnant women was 1.4% in European studies. Correct dating of maternal primary infection is crucial. An “Expert system” based on biomarkers (IgG, IgM, and IgG avidity) was developed to confirm or exclude a primary infection in the first trimester or periconceptual period, and to date the primary infection. CMV IgG Avidity II Vidas is the best assay for assessing the progressive maturation of IgG as a function of the time elapsed since primary infection. In their studies, maternal primary infection in children with cCMV sequelae occurred before 11 weeks in all cases and before 8 weeks in 89% of cases [27]. Prevention of cCMV primary infection relies on hygienic measures such as avoiding contact with toddlers’ body fluids, particularly saliva and urine.

Next, Dr. Leruez-Ville commented on how antiviral studies in pregnancy may inform the vaccine discussion. A randomized controlled trial in Israel showed that administration of valacyclovir at a dose of 8 g/day reduces vertical transmission rate by 71% in women with primary infection acquired during the first trimester of pregnancy [28]. A meta-analysis showed valacyclovir decreased vertical transmission by 64% at amniocentesis and 66% at birth [29]. Risk factors for cCMV in seropositive pregnant women are not well-established: association with caring for young children, younger age, foreign nationality or low income, concomitant maternal medical condition, and twin pregnancy all show mixed results. Globally, vertical transmission in seropositive women with active CMV shedding transmission rates is low (1.4%); transmission after re-infection may be higher. Except for the immunological parameters noted above (IgG, IgM, IgG avidity), there are no clinically available tools validated to identify pre- or post-immune women at risk of transmitting CMV to their fetus: serology is not useful in the pre-immune setting, and the positive and negative predictive values of CMV PCR in blood, urine, saliva, or vaginal secretions are unknown.

Key Points:

- The epidemiology of CMV infection in seronegative pregnant women, as well as the risk factors for infection, are both well known. Prevention of primary infection by hygienic measures is feasible, and detection and ascertainment of the timing of primary infection is possible, at least in some cases, with serology. Prevention of vertical transmission is feasible with these measures.
- CMV infection in seropositive pregnant women remains poorly understood. Risk factors require further study, the efficacy of prevention by hygienic measures remains to be documented, and identification of women at risk of cCMV, as well as prevention of vertical transmission, may be difficult.

**Speaker 7: Dr. Dana Wolf**

Dr. Wolf has shown the utility of prenatal and neonatal CMV screening in Israel. In addition to discussing this work, she presented data regarding a decidual infection system developed to model placental CMV infection [30]. This model allows the investigation of local immune protection against CMV transmission at the authentic maternal–fetal interface. Application of the decidual infection model for studies of CMV transmission and defense has generated a useful model to study placental CMV infection in cell culture. Decidual-tissue resident memory T cells protect against nonprimary CMV infection at the maternal–fetal interface and mount a rapid IFN-γ response to viral challenge [31]. In patients, this group has also identified prognostic biomarkers of cCMV infection disease severity by performing proteomic analyses of the mid-gestational amniotic fluid. In particular, they noted a group of proteins that were immune–inflammatory mediators. These results [32] suggest pathways linking aberrant inflammation at the maternal–fetal interface with the development of CMV-related fetal brain damage. Chemerin (encoded by the gene *Rarres*2, an adipokine and chemoattractant involved in inflammation, adipogenesis, angiogenesis, and energy metabolism) is a protein with regulatory roles in immune and metabolic processes and may be a biomarker for symptomatic cCMV disease. Similarly, Gal-3BP (a multifunctional immunomodulating glycoprotein) has also been identified as a potential biomarker for the prediction of symptomatic vs. asymptomatic status in the context of cCMV infection in neonates.

In the newborn screening arena, Dr. Wolf has also tested the utility of testing pools of saliva from newborns for cost-efficient newborn screening for cCMV infections, and presented data on how this may be a useful approach proposed to increase the through-put of universal neonatal cCMV testing while saving resources. Her research group tested this successfully in several hospitals in Jerusalem [33]. For pools demonstrating positive results, individual newborns can be further tested for confirmation of cCMV status. Data derived from this large-scale implementation project demonstrated the feasibility and potential benefits of pooled saliva testing to enhance the efficiency of this universal cCMV screening approach.

Key Points:

- In a cell culture model, the placenta can be demonstrated to mount an immune response to CMV infection, and this model system may enhance a mechanistic understanding of the impact of placental CMV infection on cellular responses, gene expression, synthesis of pathogenic proteins, the cytokine milieu, and subsequent injury to the developing fetus.
- There are biomarkers that have been identified that may play important roles at the maternal–fetal interface and that may potentiate the development of CMV-related sequelae in the fetus.

**Speaker 8: Dr. Suresh Boppana**

Dr. Boppana spoke about the impact of maternal immunity on infant cCMV outcomes. During a pregnancy, maternal primary infection induces CMV seroconversion, as well as positive CMV IgG and IgM responses, and a low IgG antibody avidity index. Primary CMV infection is associated with intrauterine transmission rates of 30–40% [34], varies with gestational age [20], and accounts for 25% of cCMV infections in the United States [35]. Non-primary infection (acquisition prior to pregnancy) typically produces a serological profile consisting of a positive CMV IgG, negative IgM, and a high IgG avidity index. Non-primary infections are associated with an intrauterine transmission of ~1%. Since most women of child-bearing potential in the world are CMV-seropositive, infants born to women with pre-conception immunity account for the majority of cCMV cases globally.

Dr. Boppana noted that the data are somewhat conflicting regarding the impact of maternal immunity on cCMV disease at birth and long-term outcomes. Earlier studies suggested symptomatic infection and long-term outcomes are worse in the primary maternal infection group [36]. Re-evaluation of outcomes according to type of maternal infection shows that both primary and nonprimary infection can induce sequelae [37]. In the clinic, we do not typically know if a cCMV infection is due to a primary or recurrent maternal infection. In highly immune populations, secondary (recurrent) infections may be the leading cause of cCMV. In a study by Ahlfors et al., it was estimated that 21–63% of cCMV cases were caused by a secondary maternal CMV infection [38]. The implications for how a CMV vaccine would be implemented into clinical practice, and the attendant burden on a vaccine to prevent a non-primary maternal CMV infection, are clear, as outlined by Dr. Boppana, with a need for vaccination not only to prevent primary infection but also to prevent re-infections. An understanding of how this would be incorporated into both expert recommendations and clinical practice is a challenge.

Key Points:

- cCMV infection in an infant can be due to either primary or secondary infection during a mother’s pregnancy, and in most cases, it is not known whether the infection was primary or secondary.
- Both primary and secondary maternal infections can result in symptoms and sequelae in the congenitally infected infant.

**Speaker 9: Dr. Camille Kotton**

Dr. Kotton spoke about CMV vaccination in transplant patients. The risk for CMV acquisition among transplant patients is defined by the donor and recipient CMV serologic status, with the highest risk being conferred in the setting of seropositive bone marrow recipients with seronegative donors; or seronegative SOT recipients with positive donors (D+/R−) [39]. Transplant patients receive CMV prophylaxis (typically 3–6 months after transplant) or pre-emptive CMV treatment (the monitoring period is usually once weekly for 12–18 weeks; if CMV DNA is detected then patients are treated until CMV is cleared). Both strategies are quite effective and highly used [40]. Most programs in the US use prophylaxis regimens; however, there are still high rates of CMV disease in SOT recipients, including late-onset CMV disease after discontinuation of prophylaxis. Per the international CMV guidelines for CMV in solid organ transplantation, the expert panel responsible for these recommendations was of the opinion that given the high frequency of disease in D+/R− transplant recipients, vaccines should be evaluated specifically in this group [41]. Vaccination may also reduce the burden of disease or impact in seropositive patients, and vaccine studies should include an evaluation of both humoral and cellular immunity when applicable, as well as an evaluation of the longevity of responses.

Dr. Kotton noted that comparisons between CMV in transplant recipients and cCMV have nuances such that a vaccine that demonstrates effectiveness in one population may not confer protection in the other, but expressed optimism that cCMV vaccine studies will inform and direct studies in transplant patients. There are five recent or current transplant CMV vaccine trials that have been conducted, two of which are still ongoing (Table 2).

Key Points:

- CMV causes significant morbidity in organ recipients, including late-onset CMV after prophylaxis.
- Given the high frequency of CMV disease in CMV donor-positive, recipient-negative organ transplant recipients, vaccines should be especially targeted for this population.
- CMV vaccines in transplant patients have not yet demonstrated sufficient success to allow licensure, but the gB/MF59 demonstrated encouraging results in SOT patients, and there are several novel CMV vaccine candidates currently under study.

**Speaker 10: Dr. Caleb Skipper**

Dr. Skipper discussed the indirect effects of CMV on human immunodeficiency virus (HIV) disease. The proportion of people with advanced HIV disease with CD4+ counts <200 remains constant in South Africa despite expanding efforts to increase access to treatment. CMV viremia in itself is a risk factor for advanced HIV disease. CMV viremia in HIV-infected patients with co-infection with *M. tuberculosis* shows a survival hazard ratio worse in those without viremia. High-magnitude CMV viremia is associated with developing tuberculous disease, and higher CMV IgG levels are also associated with active TB disease across a 10-year study period [46,47]. Studies of the immunopathogenesis of CMV in the setting of HIV co-infection, using in vitro models, demonstrate that TNF-a induces NF-kB, which then stimulates the IE promotor to potentiate the viral replicative cycle [48]. Dr. Skipper also pointed out that CMV produces an IL-10 homolog (UL111) which binds IL-10 receptors. IFN-γ is critical for the control of opportunistic mycobacterial infections, as demonstrated in persons with IFN-γ receptor mutations. Higher baseline IFN-γ concentrations in the cerebral spinal fluid are associated with better fungal clearance and improved survival in cryptococcal meningitis [49]. A phase III RCT of recombinant IFN-γ + antiretroviral therapy (ART) versus ART alone suggested a trend toward a decrease in HIV-associated opportunistic infections, but did not meet statistical significance. Adjunctive IFN-γ immunotherapy has shown benefits for the treatment of HIV-associated cryptococcal meningitis, although the specific contribution of CMV to the disease was not evaluated [50].

Dr. Skipper noted that CMV viremia may be a modifiable risk factor in the development of AIDS and/or HIV-associated opportunistic infections. It was noted that there are several CMV vaccination trials in populations with HIV, and several vaccine trials using CMV as a vaccine vector for prevention of HIV, exploiting an attenuated CMV vector engineered to elicit effector memory T cell responses to HIV targets. In addition to the evaluation of CMV antivirals, future CMV vaccine studies are warranted in patients with advanced HIV disease.

Key Points:

- CMV viremia is associated with mortality in advanced HIV disease, particularly in those with concomitant opportunistic infections.
- CMV may be imparting its deleterious effects via modulation of the host immune system: an impairment of the Th1 helper T cell pathway may be driving the poor outcomes seen in TB or cryptococcus co-infected persons.
- Suppression of CMV replication via vaccination or pre-emptive therapy may restore a protective IFN-γ driven Th1 immune response, preventing excess mortality from AIDS-related opportunistic infections.
- An ideal vaccine candidate would likely focus on eliciting T cell responses, with specific targeting of CMV immune modulation gene products representing additional plausible mechanistic targets.

## 3. Session 2: CMV Virology and Vaccine Targets (C. Otero and C. Crooks)

In this session, the speakers shared their work on a range of CMV vaccine targets. Dr. Michael McVoy introduced the topic by giving a comprehensive review of current viral targets and vaccine strategies and concluded by highlighting the need to explore “non-conventional” viral targets and mechanisms. Subsequent speakers each highlighted one of these “non-conventional” methods, including structure-based antigen design (Dr. Jason McLellan), identifying viral antibody targets that promote the Fc-effector function of ADCC (Dr. Richard Stanton), and targeting viral immune evasion proteins (Dr. Hartmut Hengel). Drs. McVoy, McLellan, Stanton, and Hengel highlighted the need to learn from our experiences in CMV vaccine development and explore new options for vaccine antigens and immunologic targets.

**Speaker 1: Dr. Michael McVoy**

Dr. McVoy provided a comprehensive overview of viral targets and vaccine candidates, which is a complex topic for CMV because there are many potential options among over 165 genes. Operating under a long-time predominant assumption in the field that neutralizing antibodies are key for vaccine efficacy, Dr. McVoy noted that we could focus our efforts on mediators of viral entry, but this is still not simple. CMV infects several different cell types (e.g., fibroblasts, epithelial cells, and myeloid cells) and uses a multitude of glycoproteins and protein complexes as entry mediators, so Dr. McVoy posed the tough question of which are the most important. Prior work has focused primarily on gB and the PC (gH/gL/UL128/UL130/UL131). gB is a key player in membrane fusion during viral entry, so antibodies against gB can be broadly neutralizing across cell types. However, gB-targeting responses have not demonstrated sufficiently potent neutralization to confer adequate vaccine-mediated protection. This has also been the case for the gH/gL complex, at least in animal models (no targeted gH/gL subunit vaccine has undergone efficacy testing in humans). The pentamer, on the other hand, is a known mediator of epithelial, endothelial, and myeloid cell tropism and a target of very potent neutralizing antibodies, with the critical drawback that these antibodies do not prevent CMV entry into fibroblasts.

Recent gB/MF59 (Sanofi) and V160 (Merck) vaccines provide a baseline efficacy of 40–50% against CMV acquisition and useful milestones upon which future candidates can improve. The gB/MF59 vaccine is a gB protein subunit vaccine adjuvanted with MF59, a squalene emulsion adjuvant. Neutralizing antibodies preventing infection of fibroblasts specifically were comparable to those encountered in CMV-seropositive individuals, which is often considered the minimum threshold for vaccine immunogenicity since natural immunity is not completely protective against re-infection or key disease outcomes. However, neutralizing activity against epithelial cell infection was limited. Interestingly, the partial protection observed was associated with CMV-specific antibody-dependent cellular phagocytosis (ADCP) and with ADCC, rather than neutralizing antibodies. The V160 vaccine is a replication-deficient pentamer-intact whole virus vaccine that improved upon an earlier whole virus vaccine candidate using the Towne strain of CMV, which was a pentamer-deficient live-attenuated vaccine. Due to the exclusion of the pentamer, Towne elicited limited neutralizing antibodies against epithelial cell infection, although fibroblast-specific neutralizing antibodies were comparable to CMV-seropositive individuals. However, this vaccine resulted in no protective efficacy in preventing primary infection of mothers with CMV-shedding children. V160, on the other hand, elicited neutralizing antibodies protecting both fibroblasts and epithelial cells. This vaccine conferred a 42% efficacy in preventing primary infection [26]. With regard to current vaccine trials, gB and pentamer remain the primary vaccine immunogens as Moderna continues a phase III clinical trial of mRNA-1647, based in part on the age-old premise that neutralizing antibodies are key for vaccine efficacy. Phase II studies using the and mRNA-1647 vaccine have demonstrate impressive neutralization against epithelial cell infection, with titers approximately 20-fold greater than those encountered in CMV seropositive individuals. However, Dr. McVoy brought up an important caveat that CMV’s immune evasion mechanisms are diverse and extensive [51], and short of sterilizing immunity, CMV is likely to elude even hyper-immunogenic gB and pentamer targeting vaccines due to this broad and remarkably effective immune evasion. Therefore, exploration of “non-conventional” targets and/or mechanisms is warranted.

Professor McVoy specifically suggested several key areas of research that may yield the greatest advances in vaccine development, some of which were covered in more detail in subsequent presentations:

1. Optimizing gB. As a critical player in viral entry into all CMV-permissive cell types, it is no surprise that gB has been the most popular focus of vaccine development. However, gB has largely been utilized in its post-fusion conformation, which has understandably fallen short of eliciting the desired potency of neutralizing antibody response. Thus, more directly targeting the pre-fusion conformation of gB is a promising avenue for building upon previous efforts, with the key gap in knowledge being which epitopes are unique to the pre-fusion conformation. Now that the structure of pre-fusion gB has been solved [8], there is hope for answering this question and developing a vaccine to elicit superior gB-specific neutralizing antibodies. It is unknown whether there are current vaccine platforms that might favor the pre-fusion conformation, such as whole virus formulations, in situ protein expression (e.g., DNA, mRNA, or vectored expression), fusion with other transmembrane domains, and dense bodies.
2. Considering other entry glycoprotein complexes. gM/gN is the most abundant glycoprotein complex in the virion envelope and is thought to be important for entry into all cell types through attachment via heparan sulfate. The gH/gL/gO trimer is also important for entry into multiple cell types but may be dispensable for entry into mucosal epithelial cells. However, both of these complexes include highly polymorphic and heavily glycosylated components that present a challenge in utilizing these proteins for vaccine design [52,53].
3. Eliciting unconventional antibody responses. Complement-dependent neutralization has been demonstrated against specific epitopes in gB and gM with a bias for certain gB-based vaccines, but the mechanism, including whether these antibodies act during or after viral entry, is as-yet unclear. Fc-mediated effector responses, such as ADCP and ADCC, are additional non-neutralizing antibody responses that Professor McVoy suggested were worth exploring. These generally act post-entry against viral proteins expressed on the surface of infected cells.
4. Targeting immune evasion mechanisms. As mentioned, CMV utilizes many immune evasion mechanisms, and some of these may be viable targets for vaccine development. Dr. McVoy put forward virally encoded cytokines and decoy receptors, including viral IL-10 [54], natural killer (NK) cell evasins like MHC-I homologs [55], and viral Fc receptors [56], as suggested candidates.
5. Inhibiting cell-to-cell spread. The exact mechanisms involved in cell-to-cell spread and cell–cell fusion are not well understood, but this is a key mode of dissemination that may additionally serve to evade the antibody response.

Dr. McVoy concluded by highlighting the fact that vaccine development would benefit greatly from an expanded understanding of the molecular biology and cell tropisms of CMV in vivo.

**Speaker 2: Dr. Jason McLellan**

Dr. McLellan discussed his work studying the pre-fusion confirmation of gB, highlighting the recent success of the pre-fusion F RSV vaccine [57], which elicits potently neutralizing antibodies against epitopes that are unique to the pre-fusion conformation [58]. However, McLellan noted the complexity of CMV gB compared to RSV F as these are different classes of fusion proteins with much greater change occurring between pre- and post-fusion states with gB [59]. Thus, the stabilization methods involving disulfide bond and cavity-filling mutations used for the RSV F protein did not work for gB. McLellan utilized several different strategies individually and in various combinations, including the C246S amino acid substitution, removal of the furin cleavage site, and interprotomer disulfide bonds. His work has identified interprotomer disulfide bonds as key to the stabilization of CMV gB in a pre-fusion-like conformation.

Despite developing a stable, soluble pre-fusion antigen, initial mouse immunization data does not suggest a benefit of a pre-fusion or pre-fusion-like gB-specific antibody response in neutralizing AD169, a pentamer-deficient strain of CMV, on fibroblast cells. Despite this disappointing initial result, the enthusiasm in the field for pre-fusion gB as a vaccine immunogen has not diminished. Future efforts will include additional immunization studies, antigen optimization, and monoclonal antibody isolation and characterization.

**Speaker 3: Richard Stanton**

Dr. Stanton shared his work identifying and characterizing viral targets of ADCC. As previously noted by Dr. McVoy, CMV is highly immune evasive, and Dr. Stanton specifically pointed out CMV’s ability to modulate antigen presentation, escape from the lack of self-recognition signals, and down-regulate activating NK cell receptor ligands. Many immune evasion mechanisms do not support virus replication, which is evident in the mutations that accumulate as the virus is passaged in cell culture without those immune pressures [60,61]. For example, clinical isolates of CMV spread in cell culture even in the presence of neutralizing antibodies, while passaged lab strains are more sensitive to neutralizing antibodies. This observation fits with what we know of CMV in its preference for cell-to-cell spread and suggests that antibody effector functions like ADCC that target infected cells rather than free virus may be more effective in the control of infection in vivo.

As was highlighted in Dr. Klaus Früh’s work presented in a later session, NK cells are critical to the natural control of CMV, particularly in primary infection, as individuals with NK cell deficiencies experience more severe disease. Additionally, CMV encodes a large number of NK cell antagonists to combat and modulate the NK cell response, further supporting the notion of their importance in containing CMV [55]. NK cells are more abundant in CMV-seropositive individuals and are of a phenotype that is much better at performing ADCC.

Stanton’s group has worked to characterize the antigenic targets of NK cell-mediated ADCC. He noted that ADCC activity peaks at 48 h post-infection and stabilizes at 72 h post-infection. Profiling of the plasma membrane over the course of infection identified multiple cell surface proteins that are expressed in three waves, during the early, middle, and late stages of the viral life cycle. Focusing on proteins that are uniquely expressed during the middle phase of infection at peak ADCC activity, Stanton identified two membrane proteins—UL16 and UL141—that are key viral targets of ADCC, which have been shown to mediate protection against CMV cell-to-cell spread [62].

Further study of these proteins and ADCC function has identified additional challenges to the application of these findings but also some solutions. First, studies using monoclonal antibodies developed against UL16 and UL141 revealed that they are expressed at low levels on the cell surface, which may explain why initial antibodies elicited against these proteins in natural infection are not very potent. Furthermore, the isolated monoclonal antibodies against UL16 and UL141 activated ADCC well against recombinantly expressed antigens but not against CMV-infected cells, but modification of the antibodies resulted in ADCC induction as well as induction of IFN-γ and TNFα. Furthermore, combinations of antibodies improved ADCC induction. However, the level of ADCC activation varied widely with different NK cell donors, so the antibodies themselves are not the only players we need to consider in thinking of strategies aimed toward developing ADCC-inducing vaccines or therapeutics.

Dr. Stanton closed by emphasizing the good news that ADCC seems to be a critical function in controlling CMV spread, and this function is more specifically targetable because the identified antigen targets of ADCC differ from the targets of neutralization. In addition to recent work implicating ADCC function in protection from congenital infection, higher levels of antibodies against UL16 also correlated with improved outcomes in the congenital setting [63]. However, this somewhat opposes in vitro findings that UL141-specific antibodies were more effective in inducing ADCC and begs the question of how many ADCC-driving antigens we should target. Future work highlights the need to identify the number of epitopes needed to effectively engage ADCC and ways to overcome human diversity in NK cell responses.

**Speaker 4: Dr. Harmut Hengel**

Dr. Harmut Hengel presented his work on CMV viral Fc gamma receptors (vFcγRs) as a potential target for vaccine candidates. vFcγRs are surface glycoproteins that bind to the Fc region of IgG and have been shown in vitro to inhibit FcγR activation by host effector cells [64]. Multiple other herpesviruses, including murine CMV (MCMV) and both herpes simplex viruses (HSVs), carry a vFcγR [56]. Studies of MCMV have demonstrated a clear fitness deficit with deletion of fcr-1/m38 and also delayed clearance in the salivary gland in the absence of CD16 (FcγRIII), which is the FcγR most commonly associated with ADCC function. However, human CMV is unique, insofar as it expresses multiple vFcγRs whose only known function in the viral life cycle is in playing a role in the evasion of humoral immunity, as each of the other identified vFcγRs in the non-human CMVs performs other functions beyond immune evasion. FcγRs serve as immunologic bridges, linking disparate components of the immune system together: innate to adaptive and humoral to cellular. As we heard from other speakers in this session, Fc-mediated effector functions, such as ADCC, are critical anti-CMV responses. Thus, targeting this viral immune evasion strategy could allow for antibodies against other targets to more efficiently clear virus.

Dr. Hengel has developed very useful tools for studying these proteins, specifically a panel of cell lines that each express a chimeric FcγR with the CD3 cytosolic domain, which induces IL-2 secretion upon activation of this receptor. The level of IL-2 in the culture supernatant can be measured by ELISA and used as a surrogate measure of FcγR engagement [65]. This novel reporter assay has been instrumental in the seminal work validating the immune evasion function of vFcγRs in vitro and elucidating the mechanisms behind this function. Critically, this work has shown that the CMV vFcγRs are not redundant as they act by different but cooperative mechanisms [66].

While Dr. Hengel has found that CMV hyperimmunoglobulin products like Cytotect have few antibodies targeting vFcγRs, suggesting that these proteins are not particularly immunogenic in the setting of CMV infection, a subset of monoclonal antibodies targeting vFcγRs produced through hyperimmunization of mice do block their immune evasion functions. This supports the notion that targeting these proteins through immunologic interventions can rescue these effector functions for a more effective antiviral immune response. Dr. Hengel is currently working to further characterize these monoclonal antibodies and their functional features to develop and refine vFcγR-specific antibody-based therapeutics.

Enthusiasm for targeting these proteins is further enhanced by recent evidence from Dr. Hengel’s team that has suggested a role for vFcγRs in transplacental transcytosis of CMV, as well as through evidence demonstrating direct impairment of B cell responses mediated by solubilized gp34 binding to B cell receptors, thereby preventing plasmablast formation and antibody secretion. Future work will include studying the recently identified rhesus macaque CMV (RhCMV) homologs of the vFcγRs to develop interventions, as the rhesus macaque model represents a uniquely translatable model system in which we can evaluate the efficacy of these interventions in the prevention of congenital CMV infection.

## 4. Session 3: Immune Correlates of Protection (C. Crooks, C. Otero)

In this session, speakers described techniques and models to identify immune correlates of protection against CMV. These talks underscored the complex CMV immune landscape and the need to look at all aspects of the humoral and cellular immune response. Dr. Daniele Lilleri reviewed his work examining immune correlates in primary and non-primary infection in human cohorts, highlighting the role of non-neutralizing antibodies. Examining samples from vaccinees from the most successful CMV vaccine to date, gB/MF59, Dr. Paul Griffiths highlighted his work in identifying a novel antigenic domain of the entry glycoprotein gB, which correlated with protection by preventing cell-to-cell spread of CMV. Concluding the session, Dr. Klaus Früh discussed his work in the non-human primate model to identify both the viral genes and the rhesus macaque immune responses required for RhCMV re-infection. All three speakers highlighted the need to identify novel epitopes—such as gB antigenic domain (AD)-6 and induction of unconventional T cell responses—and the importance of engaging in studying non-neutralizing antibody responses such as ADCC. Speakers also highlighted the need to look at markers of protection other than the prevention of infection, such as the prevention of disease, since this may be too high a bar to clear for a CMV vaccine.

**Speaker 1: Dr. Daniele Lilleri**

The majority of CMV infections are non-primary infections; however, most immune correlate studies focus on primary infection. Primary infections carry a 10x greater risk of transmission from mother to fetus when infection occurs during pregnancy; however, due to the greater overall number of non-primary infections, more cases of cCMV occur in non-primary as compared to primary infection. Dr. Lilleri first reviewed studies of primary infection that revealed IgM and IgG binding do not appear to correlate with the risk of vertical transmission. Although IgG binding itself did not correlate with transmission risk, IgG avidity that increases rapidly after infection was paradoxically associated with an increased, not decreased, risk of maternal–fetal transmission. However, early development of antibodies to the PC was associated with a decreased overall risk of vertical transmission in the context of primary maternal infection. This is consistent with a model of transmission risk that is correlated with higher, earlier viral replication driving these stronger and more rapid immune responses. In contrast, rapid control of viral replication is associated with reduced risk of transmission.

Because of the complexity of CMV immune responses, Dr. Lilleri and colleagues sought to test whether deep learning models could be used to predict congenital transmission in pregnant women with primary CMV infection. In 65 transmitters and 60 non-transmitters, antibody profile data was characterized for several antigens including gB and the PC. Over 100 functions, including neutralizing titers, ADCC, and ADCP, were entered into this model. The deep learning model was highly successful, with 100% accuracy at predicting transmission in primary infection. In this model, removing IgM and IgG binding had limited effects on the accuracy of the model, but IgG subclass distribution and antibody binding to Fc receptors were important for performance of the model. Importantly, the model was robust to sampling time and timing of infection in gestation.

In addition to the humoral response, Dr. Lilleri reviewed what is known about T cell kinetics in women with primary CMV infection. During primary infection, there is a low frequency of CD4+ and CD8+ T cells expressing IL2 and IL7. Approximately one year after infection there is a significant increase in the memory T cell population. Critically, development of CD4+ memory T cells is associated with reduced viral replication and transmission, highlighting its importance in controlling non-primary infection. Dr. Lilleri concluded by highlighting that T cell differentiation is a long and slow process and that it likely takes years for this response to fully develop.

Studying non-primary infection is more challenging due to the difficulty in identifying new infections in human cohorts. To address this, Dr. Lilleri and colleagues enrolled a prospective cohort of pregnant women and their neonates to characterize the immune response in mothers that did and did not transmit CMV to their infants [67]. This study revealed several key immune responses associated with cCMV. First, higher neutralizing antibody responses correlated with a higher transmission risk, whereas lower ADCC functions were correlated with a higher transmission risk. This is consistent with previous work from Semmes et al. that highlighted the importance of ADCP and ADCC in protection against transmission in a cohort of cord blood donors and their matched infants [9]. Consistent with the work described above, lower memory T cells were associated with an increased risk of transmission. Dr. Lilleri concluded by highlighting that the challenge of studying non-primary infection is the variance in infection time; however, work to determine the time since infection suggests that women with a more recent infection had a higher risk of transmitting CMV.

Key Points:

- There are shared correlates of protection in primary and non-primary infection, with an inverse correlation observed between the magnitude of neutralizing antibody titers and vertical transmission risk. The magnitude of the ADCC response may be associated with protection against transmission events observed in both primary and non-primary infection.
- Machine learning can be successfully utilized to predict transmission based on an extensive panel of immune parameters.
- T cell responses to CMV are slow to develop, with a fully differentiated T cell response taking years to develop after a primary infection; robust CD4+ memory T cell responses have been associated with reduced replication and transmission and are worth emphasizing in vaccine development.

**Speaker 2: Dr. Paul Griffiths**

While there are currently no licensed vaccines for CMV, the gB/MF59 vaccine showed 43–50% efficacy across different populations in phase II clinical trials [3,4,45]. Dr. Griffiths presented his work studying the immune response to the gB/MF59 vaccine in a phase II trial in SOT recipients [45]. Looking at the DNAemia in these vaccinees, those who received that vaccine had a shift in their viral load peak earlier in infection with an overall reduction in the duration of DNAemia. This shifted viral load trajectory is similar to what is seen in seropositive individuals re-infected with CMV. This highlights that the vaccine functioned with similar, but not superior, efficacy to natural immunity, suggesting that there may need to be a different approach to vaccination in seropositive individuals than in seronegative individuals. Sterilizing immunity may be an impossible target for CMV vaccination, and this work suggests that reduced CMV viral loads could be an alternative target for vaccines to prevent transmission of CMV.

In analyzing humoral immune data from SOT recipients in phase II clinical trials of the gB/MF59 vaccinee, IgG binding to the vaccine antigen—the Towne strain of gB—has been identified as a correlate of protection. However, in work by Dr. Griffiths and others, neither neutralization, ADCC, nor binding to a specific antigenic domain (AD 1–5) of gB was correlated with protection in these trials (emphasizing that these were in transplant patients, and not pregnant persons), suggesting that the gB/MF59 vaccine offers protection via a distinct mechanism. Linear peptide scanning of gB revealed that a novel antigenic domain, termed AD-6, was associated with protection in seronegative vaccinees [68]. AD-6 is a highly conserved, 50 amino acid polypeptide within protein domain 5 of gB. Responses to AD-6 were present in more than 70% of vaccinees but were only present in less than 5% of naturally infected individuals. Because of its position within domain 5 of gB, this polypeptide is largely obscured when gB is in its pre-fusion conformation on the surface of the virion suggesting that it offers protection via a non-neutralizing mechanism. Dr. Griffiths and colleagues determined that antibodies to AD-6 provided protection by blocking cell-to-cell spread of CMV.

Looking forward, Dr. Griffiths suggested that it will be important to examine whether other vaccines similarly induce AD-6 responses and whether these responses also inhibit cell-to-cell spread of CMV. He concluded by reviewing the different eras of CMV vaccine development, and by observing that the next generation of vaccines will likely need to be informed by elucidation of cryptic epitopes such as AD-6, and by an enhanced understanding of potentially novel mechanisms of anti-viral immunity.

Key Points:

- Reduction in CMV DNAemia is a potential alternative to sterilizing immunity as an CMV vaccine study endpoint.
- AD-6, a novel antigenic domain of gB, blocks CMV cell-to-cell spread and this protein has been identified as a potential vaccine-induced correlate of protection in SOT recipients.
- Future vaccine development must be informed by novel mechanisms of anti-viral immunity.

**Speaker 3: Dr. Klaus Früh**

While human cohort studies and clinical trials of CMV vaccines provide crucial insights into the immune response to CMV, utilizing animal models complements this work by identifying specific viral determinants of infection and re-infection. To model virological determinants of the immune response to CMV, Dr. Früh has worked with the OHSU National Primate Research Center to help pioneer the rhesus macaque model of RhCMV. Because most rhesus macaques are naturally infected with RhCMV, studies that require seronegative macaques are often more costly and challenging due to the need to maintain a RhCMV seronegative colony. However, previous work by Dr. Früh and colleagues has demonstrated that, despite detectable T cell and antibody responses, seropositive macaques are regularly reinfected with RhCMV when experimentally challenged.

Infection dynamics of RhCMV in this challenge model are monitored by performing qPCR on known simian immunodeficiency virus (SIV) antigens that have been inserted into the RhCMV viral genome. Long lasting effector memory T cell responses to these inserted SIV antigens are detected, which supports the idea that macaques are productively infected, and these re-infections stimulate a novel immune response. While viral detection in plasma and immune responses indicate productive re-infection, re-infected macaques have reduced virus present in urine and saliva suggesting that there is limited disease in this model.

Dr. Früh and colleagues have previously used a panel of deletion mutants lacking genes that were determined to be disposal for infection in vitro to understand which viral genes are required for infection. All deletion mutants were able to reinfect macaques except for one construct that had two genes determined to be essential for re-infection: a UL131A homolog, part of the PC, and thought to have an undefined role in immune evasion in rhesus macaques; and Rh159, a UL148 homolog that has an NK-cell evasion function, similar to UL16, that downregulates the expression of NKG2D. Rh159 is required not only for re-infection, but also for primary infection, except when CD8+ T cells including NK cells, are depleted. This suggests that evading NK-mediated immunity is required to establish RhCMV infections [69].

After identifying which immune responses needed to be evaded to establish infection, Dr. Früh and colleagues looked at the function of the non-essential genes in the context of infection in vivo. These studies used a double-deletion mutant that lacked UL128-UL130 (a component of the PC, and known to inhibit unconventional MHC-E and MHC-II restricted CD8+ T cell responses) and UL146-UL147 (viral CXC-chemokine like proteins). This double mutant results in lower infection in vivo [70]; however, the ratio of infected macrophages and endothelial cells does not change in infection with the double mutant even though this is PC-deleted virus, which is thought to restrict replication in endothelial and epithelial cells [71]. In looking at single mutants of UL146 and UL128-UL130, the UL146 deletion mutant was significantly more attenuated in vivo than the UL128-UL130, suggesting that an intact PC may not be required for in vivo infection with RhCMV [72].

Other work by Dr. Früh and colleagues has looked at using RhCMV itself as a vector. This vector has been successful in protection against SIVmac293 by eliciting unconventional MHC II and MHC-E restricted CD8+ T cells. Recent work tested a single-cycle RhCMV vaccine by deleting late genes [73].

Collectively, this highlights the fact that CMV has multiple immune evasion mechanisms that need to be overcome to prevent infection. Natural CMV immunity generally provides protection against disease in most individuals but does not provide protection against infection. This is consistent with the point that Dr. Griffiths made about the need to evaluate the most appropriate endpoints for CMV vaccine trials and whether sterilizing immunity should be the goal. Dr. Früh made a compelling argument that prevention of infection is too high of a bar to clear for a CMV vaccine and that the focus should be on prevention of disease. Because natural T cell responses do not predict reinfection risk, future vaccine designs should look at unconventional T cell responses or antibody responses that target CMV immune evasins.

Key Points:

- Evasion of NK cells is required for primary infection, but evasion of CD8+ cells is necessary for non-primary infection.
- Deletion mutants are powerful tools to study the immune response to RhCMV in vitro and in vivo and can reveal novel immune mechanisms such as unconventional T cell responses.
- Prevention of infection may be too high of a bar to clear, and therefore prevention of CMV disease could be a more suitable goal.

## 5. Session 4: CMV Vaccines in Development and in Clinical Trials (H.-Y. Wang, K. Karthigeyan)

The CMV vaccine field has seen the development of many candidates of late, with several new platforms, including the mRNA-LNP platform similar to that utilized for the SARS-CoV-2 vaccine (Moderna Vaccines), a virus-like particle (VLP) vectored vaccine (VBI Vaccines), nanoparticle vaccines, adjuvanted protein subunit vaccines, and MVA-vectored vaccines.

In this session, Dr. Rajiv Khanna summarized work on the ISS-1018 adjuvanted CMV vaccine, while Dr. Dave Anderson discussed VBI Vaccines’ enveloped virus-like particle (eVLP) vaccine. Dr. Ann Arvin from Vir Biotech presented the rationale for using CMV as a T cell vaccine platform, and reviewed candidates based on this platform that are currently in clinical trials. Dr. Laurent Perez summarized work on a trimeric CMV nanoparticle vaccine based on AD-5 of the gB molecule, and Dr. Bodo Plachter discussed his work characterizing the immunogenicity of CMV dense bodies as a potential vaccine candidate. Dr. Hannah Alsdurf highlighted GSK’s efforts to foster diversity and inclusion in clinical trials of CMV vaccines.

Two speakers (Drs. Don Diamond and Ajit Limaye), spoke about CMV vaccine development using the MVA delivery platform. Drs. Diamond and Limaye’s presentations focused on the CMV Triplex vaccine, which has been tested in HSCT patients and is currently being tested in SOT population in liver transplant recipients. Dr. Kevin Russell presented the phase 2b clinical trial results for Merck’s V160, a replication-defective CMV viral vaccine. New technologies for CMV vaccines were also highlighted. Dr. Sumi Biswas spoke about using the SpyCatcher (protein partner) and SpyTag (peptide label) nanoparticle technology to deliver the CMV PC in a novel vaccine design. Dr. Gry Person introduced the AI-Immunology (TM) platform and presented how to apply this platform for CMV vaccine immunogen prediction.

Overall, several exciting vaccine candidates at various development stages were discussed. There is increased recognition of the necessity for a vaccine candidate to be able to elicit robust T cell immunity, as well as the need for more research into immune correlates of protection against cCMV.

Table 3 provides a summary of the candidates reviewed and discussed in this session, and individual platforms and candidates presented at the workshop are discussed on a speaker-by-speaker basis below.

**Speaker 1: Dr. Rajiv Khanna**

Dr. Rajiv Khanna from the QIMR Berghofer Medical Research Institute, Australia, summarized preclinical research that led to development of the ISS-1018 adjuvanted CMV vaccine, which induces polyfunctional CMV-specific CD8+ and CD4+ T cells and gB-specific B-cell responses in mice. He first commented on the current goalposts for a CMV vaccine and opined that the benchmark needs to be prevention of disease and not infection. He also advocated strongly for the inclusion of a T cell component in vaccine design, insofar as a lack of T cell responses or a reduction in T cell immunity can potentiate infection and increase the risk of CMV in diverse patient populations.

Dr. Khanna spoke about a QuantiFERON CMV blood test that is specifically designed to measure T cell immunity and allows for the measurement of competent T cell responses through assessing IFN-γ levels [74,75]. This test returns a result within a few hours and a positive test coincides with protection from CMV reactivation in transplant recipients. Dr. Khanna mentioned the Kotton guidelines on the management of CMV in SOT recipients [41], and emphasized that using the QuantiFERON blood test in conjunction with adoptive immunotherapy with CMV-specific T cells lead to complete resolution of drug-resistant CMV-associated complications in 11 of 13 solid organ transplant recipients [76]. QIMR Berghofer Medical Research Institute is now a major off-the-shelf source for T cell therapies for patients with CMV disease being increasingly used under the Therapeutic Goods Administration (TGA) special access scheme in Australia.

Dr. Khanna then spoke about the design of a two-component CMV vaccine, with each component inducing T cell and B-cell immunity [77]. The first component is a polyepitope that includes multiple CMV T cell antigens (pp65, IE-1, pp150, pp50, and DNase) that provide a global population coverage, based on matching of class I-restricted epitopes to predominant HLA alleles, of 94%. The other is a trimeric gB ectodomain, without the membrane-proximal region but with the cytoplasmic tail reattached. Trimeric gB allows access to fusion loops in domain I of gB that are critical for host cell binding. In preclinical immunogenicity studies in mice, this two-component CMV vaccine, adjuvanted with ISS 1018, induced polyfunctional and CMV-specific CD8+ and CD4+ T cell responses that were highly durable. Immunized mice also exhibited gB-specific B-cell and antibody responses. A booster on day 210 enhanced the CMV-specific antibody responses with minimal impact on T cell immunity.

Dr. Khanna also commented that the ectodomain of gB is not hidden or different between the pre- and post-fusion conformations of gB, and that a successful vaccine does not need to be in a prefusion structure, but one does need to expose the correct epitopes to trigger B and T cell responses. Dr. Khanna also emphasized the need for more research on immune correlates of protection against congenital CMV transmission.

**Speaker 2: Dr. David Anderson**

Dr. Anderson from VBI vaccines discussed an eVLP system for the development of a CMV gB vaccine, in which particles are produced in cells after expression of murine leukemia virus (MLV) viral matrix protein Gag, to express either full-length gB (gB eVLPs) or the full extracellular domain of CMV gB fused with the transmembrane and cytoplasmic domains from vesicular stomatitis virus (VSV) G protein. In this system, the antigen is delivered by a two-plasmid system: one encoding MLV Gag and the other encoding the glycoprotein construct of interest. This presentation induces potent neutralizing antibodies: for example, SARS-CoV-2 Spike expression [78] can be enhanced through this system.

The VBI CMV vaccine selected for clinical trial development was the engineered virus-like particle (eVLP) gB vaccine with the gB ectodomain fused to the transmembrane and cytoplasmic tail of the vesicular stomatitis virus G protein, designed for improved expression (termed gB-G). In preclinical studies, gB-G exhibited trimeric expression and elicited better epithelial neutralization titers compared to the full-length monomeric gB protein. A randomized, placebo-controlled phase I trial (NCT02826798) was conducted in 125 CMV seronegative, healthy adults (18–40 years) involving a three-dose regimen consisting of 2 μg of gB-G; the use of alum adjuvant significantly improved immunogenicity. Overall, this vaccine was safe and immunogenic in seronegative, healthy participants. High gB binding, avidity, and neutralizing antibody titers after three doses, as well as a response to the potently neutralizing gB epitope, AD-2, were observed in 24% of participants. In addition, 100% of vaccinees mounted fibroblast-neutralizing antibody responses while a third of the participants mounted epithelial-neutralizing antibody responses. The phase I safety and immunogenicity data were recently published [79].

Finally, Dr. Anderson presented VBI Vaccines’ proprietary new technology called mRNA-launched eVLPs (MLE) which combines the strengths of eVLP and mRNA platforms. In this approach, eVLP particles are coded in mRNA which adds manufacturing speed (an important attribute of typical mRNA expression platforms). Currently, several animal studies are ongoing to compare the expression of target antigens, including CMV immunogens, through the MLE platform, with a comparison to traditional mRNA expression alone.

**Speaker 3: Dr. Ann Arvin**

Dr. Arvin from Vir Biotech presented the rationale for CMV as a T cell vaccine platform. Their approach involves using CMV promoters to drive the expression of foreign genes, using whole-virus CMV constructs that have the UL128–131 open reading frames (ORFs) of the genome deleted. These ORFs encode constituents of the CMV PC, but also modify viral T cell responses, and deletion of this region in the RhCMV leads to a virus that induces broader, non-canonical T cell responses to more diverse epitopes—a feature desirable in a vaccine [80]. The approach leads to improved antigen expression and long-term persistence as well as higher frequencies of CD4+ and CD8+ T cells that persist without exhaustion. There is also good expression of effector memory T cells (TEMs). They hypothesize that CMV promoter-driven expression of foreign genes will confer desired and improved immunologic characteristics. This CMV-vectored vaccine technology was developed in collaboration with Drs. Louis Picker and Klaus Früh at Oregon Health & Science University (OHSU), USA.

Their CMV backbone is based on a bacterial artificial chromosome (BAC) derived from the TR3 tissue-culture passaged clinical isolate. This Vir2 backbone was made by introducing single nucleotide polymorphisms into the wild-type TR3. Caposio et al. describe in detail the construction of the TR3-BAC backbone [81]. CMV-Vir2 vectored vaccines lead to high frequencies of CD4+ and CD8+ responses directed against different proteins of the target virus, with CMV-specific T cells constituting about 10% of all cells. These responses are also sustained at very high frequencies relative to the age of the individual. A RhCMV-vectored vaccine using this approach was able to protect against *M. tuberculosis* challenge in rhesus macaques [82]. The OHSU group also showed that RhCMV vectored vaccine was protective and durable against SIV challenge in rhesus macaques [83].

Dr. Arvin also discussed Vir-1111, Vir BioTechnology’s prototype, proof-of-concept human CMV-vectored HIV vaccine, which recently finished phase I trials in healthy volunteers (NCT04725877). This vectored vaccine expressed HIV clade A Gag, a structural protein essential for HIV replication. While this prototype did not yield the desired outcomes, data from this study informed and directed subsequent development of the next-generation VIR-1388, also based on the same CMV-vectored vaccine design, again with the goal of increasing T cell immunity. VIR 1388 is currently in phase I trials testing the safety and immunogenicity of three different doses in healthy, CMV seropositive adults without HIV.

**Speaker 4: Dr. Laurent Perez**

Dr. Perez presented work on a two-component nanoparticle system vaccine, developed at the Université de Lausanne which displays a multimeric gB immunogen fused with a trimeric gB AD-5 antigen presented on self-assembling nanoparticles. This display increased gB immunogenicity as seen in preclinical studies in mice, including strong antibody binding to soluble gB and gB on the cell surface, and neutralizing antibody titers comparable to levels in convalescent plasma.

Dr. Perez first laid out the rationale for choosing AD5 as an antigen for a nanoparticle vaccine candidate, which is based on mouse immunogenicity work characterizing the neutralizing abilities of the individual ADs of gB. Mice were immunized with the individual antigenic domains or the gB ectodomain as a control. While all ADs elicited comparable plasma IgG binding to the gB ectodomain and to the autologous immunogen post-immunization, AD-1, AD-2, and AD-4 did not elicit neutralizing antibodies without complement in fibroblasts and epithelial cells. AD-5 immunization elicited potent neutralizing antibodies in both fibroblasts and epithelial cells at significantly higher levels than gB ectodomain immunization. Depletion of AD-5 antibodies from sera also greatly reduced the neutralizing ability of the gB ectodomain, indicating that most of the neutralizing ability of gB is from AD-5. Dr. Perez also mentioned that there is good overlap between AD-5 in prefusion and post-fusion states of gB; hence, the conformation state of gB does not make a big difference in this system.

To present AD-5 in a vaccine, Perez and colleagues used a ferritin scaffold as well as an I5350 scaffold. The trimeric AD-5 antigen (trAD-5) was made by fusing gB AD-5 with the gB core domain and then assembling it into nanoparticles with ferritin or I5350, each displaying eight and twenty trAD5, respectively. Mouse immunogenicity studies revealed that nanoparticle display of trimeric AD5 significantly boosted antibody binding and neutralizing titers. This work was recently published [84]. During the discussion, there was interest in producing the recently discovered AD-6 domain of gB using this system, but these experiments have not yet been commenced.

**Speaker 5: Dr. Bodo Plachter**

Dr. Plachter from Johannes Gutenberg-University in Mainz, Germany, discussed the potential of CMV-dense bodies (DBs) as vaccine candidates. DBs are nonviral microbodies containing viral antigens in CMV-infected cells. These dense bodies are non-infectious particles released in large amounts from infected fibroblasts that are devoid of viral capsids and viral DNA, and can induce both cellular and humoral responses [85,86].

Vaccination with recombinant dense bodies protects immunocompetent mice against a high-dose challenge infection with MCMV. Proteome analysis revealed upregulation of interferon-stimulated genes in cells incubated with DBs, overall leading to an antiviral type I interferon response [87]. DBs are produced from CMV-infected fibroblasts through ultracentrifugation, fractionation, and ultrafiltration of the supernatant containing virus particles and dense bodies.

The DBs are made from a Towne virus BAC repaired to express PC in order to produce PC-positive DBs. This is in order to analyze the contributions of PC towards neutralizing responses elicited by a DB vaccine in comparison with PC-negative DBs. In mice and rabbit immunization studies, PC-positive DBs demonstrated higher neutralizing ability when produced in fibroblast, epithelial, and endothelial cells as compared to PC-negative DBs.

Dr. Plachter stated that the next steps are to produce clinical-grade DBs to prepare for a phase I study, for which a sponsor is needed. The production process and protocol for an eventual trial have already been set up.

**Speaker 6: Dr. Hannah Alsdurf**

Dr. Alsdurf from GSK spoke about efforts to foster inclusion and diversity in clinical trials, based on lessons from the COVID-19 pandemic that revealed an underrepresentation of racial and ethnic minorities, although these communities often had a disproportionately higher burden of disease. They identified a need for specific plans to recruit racial and ethnic minorities and to use epidemiologic data as a benchmark for assessing clinical trial diversity.

They analyzed 495 US-based trials (Phase 1–4) conducted by GSK between 2002 and 2019 and compared demographic diversity in those trials with US census and epidemiologic data. They found that a large number of trials met or exceeded census criteria for non-Hispanic white individuals. Few trials met the census criteria for Asian, indigenous, Asian American-Pacific Islanders, Latino, or multiracial individuals. Black/African Americans made up 60% of vaccinees in their phase I trials but the percentage declined in phase II trials. Details and results of this study were recently published [88].

Dr. Alsdurf spoke about GSK’s efforts in using inclusion and diversity (I and D) surveys to better understand participation in clinical trials in terms of awareness, barriers, and motivators. For instance, in a Gonorrhea clinical trial, they assessed the importance of an educational intervention in the form of an online survey to combat hesitancy and found the highest improvement in those who were marginally hesitant. They applied education in a CMV I and D survey as well. CMV seroprevalence is higher among Black and Hispanic women, and older age is also an associated risk factor. In the online survey, a CMV educational intervention to increase willingness to participate in clinical trials surprisingly had a negative effect and decreased the proportion of those willing to participate. An updated survey included more concise and simple language and highlighted risk factors for cCMV. They recruited 680 participants for the survey (16% Hispanic and 33% Black women) with the primary goal of increasing recruitment among minorities. A total of 45% of participants had children while 25% intended to have children in the future.

Their analysis of this intervention is ongoing, but their initial conclusions are that targeted messaging had a disappointingly limited impact on high-risk groups. The intervention had the strongest impact on women intending to become pregnant and those whose modalities were only marginally hesitant about participation initially. There also seemed to be an increased willingness to participate in CMV trials following this intervention. The need to leverage social media for impact, like Magic Johnson working with GSK for awareness on RSV vaccines, was highlighted. The need for tailored approaches to improve participation was also emphasized. There was considerable discussion on needing to budget enough time towards recruitment, toward the goal of improving diversity (recognizing that there are significant health disparities related to cCMV). It was also noted that there is great importance in building relationships that in turn can promote participation in future clinical trials.

**Speaker 7: Dr. Don Diamond**

Dr. Diamond spoke about the progress of Phase 2 Clinical Trials of CMV-MVA-Triplex Vaccine in the City of Hope Medical Center, California [89,90,91]. He first mentioned that antiviral prophylaxis treatment (letermovir) successfully reduced the burden of CMV reactivation but did not solve the problems such as late CMV reactivation, the number of times a person needs to be treated with letermovir, and the high toxicity of antivirals. Therefore, CMV vaccine development for transplant patients remains essential to alleviate the challenging problems caused by the antiviral treatments. Dr. Diamond opined that the MVA vector confers multiple vaccine development advantages, including safety and high attenuation, replication in cytoplasm using its own machinery so that no chromosome integration occurs, thermostability, suitability for large-scale production, and large capacity for inclusion of foreign nucleic acid. CMV-MVA Triplex (pp65, IE-1, and IE-2) generated strong preclinical data in HLA transgenic mice and human peripheral blood mononuclear cells (PBMCs) isolated from CMV seropositive individuals and transplant patients ex vivo [92]. Dr. Diamond noted that, to date, 12 ongoing or completed clinical trials have tested various iterations of MVA-vectored CMV vaccines in transplant settings, with the main goal of eliciting CMV-specific T cells with memory phenotypes. In clinical trials, CMV-MVA Triplex vaccine was also administered to donors before transplant, toward the goal of boosting CMV-specific T cell responses, which could then be, in essence, “adoptively transferred” to the immunocompromised recipients. Ongoing and future vaccine development at City of Hope focuses on broadening the properties of the MVA-vectored CMV vaccine. These include the incorporation of more antigens to cover immunodominant humoral and cellular responses; enabling strategies for both prophylactic and therapeutic approaches; and construction of vaccines using the City of Hope-owned MVA platform.

Two important questions were raised during the discussion:

*1.*  *What is the effect of pre-existing immunity to poxviruses on MVA vectored vaccines, from the perspective of lifelong immunity to smallpox?*

Answer: Individuals who had already received the vaccine still responded fully to MVA-vectored vaccines.

*2.*  *Did you stratify donors and recipients by CMV status in your study?*

Answer: The CMV status was indeed stratified, and it was definitely more difficult to recruit CMV seronegative donors. Donor/recipient mismatch made studies complicated. However, the FDA still approved combinatorial seroprevalence studies. Often the haplotype match is with siblings who are matched with regard to CMV status.

**Speaker 8: Dr. Ajit Limaye**

Dr. Limaye from the University of Washington spoke about Triplex CMV vaccine in the orthotopic liver transplant setting [93]. Between 80 and 90% of cases of CMV disease observed in liver transplant recipients are due to a D+R− (donor CMV seropositive, recipient CMV seronegative) mismatch, indicating the significance of CMV vaccine development in liver transplantation [94,95]. The phase 2 clinical trial (CTOT-44) target population is CMV seronegative adults with liver disease pre-transplant (N = 416 total, 208/arm). The trial is envisioned to be an RCT double-blind placebo-controlled study with up to 20 centers. Vaccinated individuals are given 2 doses of Triplex vaccine or placebo 28 days apart by intramuscular injection. The endpoints of the trial include safety, immunogenicity (longitudinal ELISPOT), immune correlates, and efficacy (duration of antiviral therapy). The trial was designed to target liver transplants but not kidney transplants as a good initial study because: (1) high rates of CMV disease occur after liver transplants even with current preventive strategies; (2) CMV infection in liver is associated with worse outcomes; (3) liver transplant patients receive lifelong immunosuppression therapy and thus are exposed to a lifelong risk; (4) liver is more vulnerable to hematologic toxicity of antiviral drugs compared to other organs; and (5) there is no FDA-approved prophylaxis drug for liver patients. The trial was designed to be analogous to pre-emptive therapy (PET) instead of prophylaxis, because PET decreases CMV disease and increases CMV-specific T cell immunity in response to preceding DNAemia. The immune correlates of protection suggest that multifunctional T cells and epithelial cell entry neutralizing antibody response are both potentially associated with protection from CMV disease; this would in principle be recapitulated in the CMV vaccine design anticipated for the trial. This study, known as Cytomegalovirus (CMV) Vaccine in Orthotopic Liver Transplant Candidates (or “COLT” study), will be conducted in nationally recognized liver transplant centers throughout the USA, funded through an NIAID grant.

**Speaker 9: Dr. Kevin Russell**

Dr. Russell from Merck presented results from a double-blind, randomized, placebo-controlled phase 2b multi-center trial of V160, a replication-defective CMV vaccine that induces responses multiple CMV antigens, including both gB and the PC [26]. CMV PC is an important vaccine target because PC has a role in viral entry and replication in epithelial and human placental cytotrophoblasts and is therefore important for maternal–fetal CMV transmission. The V160 was designed to elicit both humoral and T cell responses similar to wild-type CMV infection. For the safety profile, V160 replication was controlled so that a productive infection/latency could not be established in vivo. V160 was given through either intramuscular or intradermal routes, and was well tolerated and induced humoral and cell-mediated immune responses compared to natural immunity in all of the trial participants. The V160 vaccine was later given to 16–35 year-old seronegative females who had direct exposure to children under 5 years old. The urine and saliva samples were collected monthly by self-collection. CMV infection was defined by the detection of non-vaccine type CMV by PCR from a single saliva or urine sample in a CMV-uninfected participant, and immunogenicity was reported. There were no safety concerns reported from the clinical trials. Regarding viral load detection, saliva was found to be a better indicator for CMV identification than urine. In terms of humoral immunity, neutralizing antibody titers elicited by 2-dose and 3-dose regimens were different, but the IgG binding to gB was similar. ELISPOT data showed that the T cell response peaked at 7 months post-immunization and continued to be detectable 24 months later.

**Speaker 10: Dr. Sumi Biswas**

Dr. Biswas from Spy Biotech (England) presented information about a newly developed nanoparticle CMV vaccine program. SpyBiotech started at Oxford in 2017. Currently, Spy Biotech has commenced programs to pursue malaria vaccine and CMV vaccine clinical trials [96,97,98]. The nanoparticle vaccine core technology is based on a combination of SpyCatcher (protein partner) and SpyTag (peptide label) and aims to elicit both humoral and T cell responses [99]. The VLP-SpyCatcher platform relies on the antigen-SpyTag to create a universal plug-and-display decorated VLP system. This platform enables the generation of a strong covalent bond between the vaccine immunogen of interest and the protein partner, with the manufacturing advantage of there being no limitations with respect to antigen size and complexity. Using this technology, SPYVLP (hepatitis B surface antigen), SPYVECTOR (adenovirus), and SPYLP01 (CMV) platforms were developed [100]. SPYVLP01 is a PC-based vaccine since PC is beneficial for included in a CMV vaccine. The SpyTag was designed to tag on the gH component of the PC. The SPYVLP01 elicited antibody responses better than protein alone, even at a small dose, and the neutralizing antibody titer was reported to be similar to that in CytoGam^®^ (2.5 mg/mL). In phase 1 clinical trials in the UK, SPYVLP01 was given to six groups, at 15 or 30 mg with and without adjuvants 500 ng alhydrogel or 50 micrograms of Matrix M.

**Speaker 11: Dr. Gry Person**

Dr. Person from Evaxion Therapeutics (Denmark) discussed strategies to use AI for CMV vaccine development and immunogen prediction (https://www.evaxion-biotech.com/, accessed on 1 October 2024). Evaxion started its AI program 15 years ago. The core AI tools at Evaxion include EDEN™, RAVEN™, and BIFORST™. EDEN™ was designed to identify protective B-cell antigens. They were trained by validated public data on recognized protective and non-protective antigens to recognize shared protective features. RAVEN™ was designed to identify functional T cell epitopes after training by viral or bacterial proteomic and genomic data and target population MHC data. RAVEN™ is able to identify T cell epitope hotspots in the entire pathogen genome or selected genes and perform HLA-matched predictions as well as pathogen variant coverage. BIFORST™ was designed to engraft T cell epitopes into B-cell antigens while retaining the conformation, but no further details were provided in the presentation. Utilizing AI tools for immunogen prediction will be extremely applicable to CMV vaccine development since CMV was reported to have >250 canonical ORFs and even >750 ORFs, which makes it difficult to identify which ORFs should be included as immunogens.

## 6. Summary and Conclusions

In summary, this Conference allowed the presentation and the review of many aspects of CMV epidemiology and immunobiology that are germane to vaccine development. The current state of knowledge of the correlates of protective immunity was reviewed, with the recognition that the immune responses preventing vertical CMV transmission may not be the same as those that are responsible for preventing or reducing CMV end-organ disease in HSCT and SOT patients. The current status of clinical trials in both settings—protection against cCMV and prevention of CMV in the transplant setting—was reviewed. The results of a phase III efficacy study of an mRNA vaccine in preventing primary CMV infection are anticipated in the coming year. The meeting organizers urge academic investigators, vaccine manufacturers, and advocacy groups to continue the aggressive study of the many novel platforms in preclinical development and clinical trials. Since CMV is the most common infectious cause of disability in children, licensure of a CMV vaccine continues to be a high priority.

## Figures and Tables

**Table 1:** Strategies to reduce the burden of cCMV.

Goal	Strategies
Prevent cCMV infection during pregnancy	Education to reduce CMV exposures among pregnant persons Vaccination (vaccines in phase I-III clinical trials)
Prevent transmission of cCMV infection to the fetus	Test for and detect primary maternal CMV infection during pregnancy Promising findings of high-dose valacyclovir treatment following 1st trimester primary infection (not recommended in the US)
Enhance detection of cCMV infection	Newborn screening and early intervention for SNHL Antiviral treatment for symptomatic infants

**Table 2:** Vaccine trials in SOT and HSCT recipients.

Vaccine Trial	Status
ASP0113 (DNA vaccine, Astellas) [42,43]	Failed in both high-risk kidney and HSCT recipients
HB-101 (LCMV vector with CMV antigens, Hookipa) in D+R− living donor kidney transplant recipients [44]	Phase 2 trial stopped due to lack of efficacy
gB/MF59 subunit vaccine effective at decreasing duration of viremia in high-risk kidney and liver transplant recipients [45]	Development did not proceed
Triplex (Multi-peptide CMV vaccine in Modified Vaccinia Ankara [MVA] vector, City of Hope)	Underway in HSCT, and starting in liver transplant patients, “CMV vaccine in Orthotopic Liver Transplant” (COLT) study, ClinicalTrials.gov: NCT06075745
mRNA-1647 CMV (Moderna) in undergoing clinical trial evaluation in an allogenic stem cell transplant recipient population to evaluate the first clinically significant cytomegalovirus infection in the period following cessation of CMV prophylactic treatment (on Day 100 post-HSCT) through month 9 post-HSCT	Currently ongoing, ClinicalTrials.gov: NCT05683457

**Table 3:** CMV vaccine candidates and key points.

CMV Vaccine Candidate	Current Stage	Key Points
EVX-V1	Target Discovery	Applying AI-Immunology (EDEN™, RAVEN™, and BIFORST™) to predict immunogen design. Extremely applicable to predict targets from the large CMV genome that encodes >750 ORFs.
CMV ISS-1018	Preclinical	Bivalent protein subunit vaccine expressing CMVpoly and gB with CpG1018 adjuvant. Studied in HLA-expressing mouse model. Induced polyfunctional CMV-specific CD8+ and CD4+ T cell response in mice in vivo. Elicited gB-specific B-cell responses in mice in vivo.
trAD5-nanoparticle (I5350/Ferritin)	Preclinical	Two-component nanoparticle vaccine expressing multimeric gB immunogen fused with trimeric gB AD-5. Used self-assembling I5350 and Ferritin nanoparticles as delivery platforms. gB AD-5 is the only AD that elicits nAb responses without the presence of complement. trAD5-nanoparticles enhanced gB-specific Ab binding and nAb titer in mice in vivo.
Dense Bodies (DBs)	Preclinical	Non-infectious microbodies with viral antigenicity. Largely released from infected fibroblasts. Recombinant DBs protected immunocompetent mice against a high dose MCMV challenge infection. DBs isolated from PC-repaired CMV elicited higher nAb titer in fibroblasts, epithelial, and endothelial cells.
SPYLP01	Phase 1	Used SpyCatcher and SpyTag nanoparticle technology to deliver CMV PC. Elicited greater Ab binding than PC protein alone. Elicited nAb titer similar to CytoGam^®^. Studied with and without adjuvants alhydrogel or MatrixM in clinical trial.
VBI-1501	Phase 1	VLP expressing CMV gB ectodomain fused with VSV-G protein transmembrane and cytoplasmic tail. Adjuvant Alum significantly improved immunogenicity. Elicited strong gB-specific Ab binding and nAb titer after 3rd dose.
CMV-MVA-Triplex	Phase 2	MVA vector encoding CMV pp65, IE-1, and IE-2. Strong preclinical data in HLA-transgenic mice in vivo and human PBMCs ex vivo. Studying in HSCT recipients and donors to evaluate safety and immunogenicity. Studying in orthotopic liver transplant patients for preemptive therapy.
V160	Phase 2b	Replication-defective CMV vaccine with repaired PC. V160-elicited humoral and cellular immune responses similar to natural immunity. CMV infection, as defined by PCR, and immunogenicity, instead of seroconversion, were monitored in the clinical trial.
mRNA-1647	Phase 3	mRNA vaccine targeting gB and PC. Robust immunogenicity in phase 2 studies. Phase 3 protection study fully enrolled. Adolescent and transplant studies planned.

## Data Availability

Original session notes obtained during the conference are available upon request from the corresponding author (M.R.S.).
